# Oral beta-lactam step down in bacteremic *E. coli* urinary tract infections

**DOI:** 10.1186/s12879-020-05498-2

**Published:** 2020-10-21

**Authors:** Stephan Saad, Neil Mina, Colin Lee, Kevin Afra

**Affiliations:** 1 Department of Medicine, University of British Columbia, 2733 Heather Street, Room C328, Vancouver, British Columbia V5Z 3J5 Canada; 2 Department of Laboratory Medicine, Fraser Health, 13750 96 Ave, Surrey, British Columbia V3V 1Z2 Canada; 3 Department of Pharmacy, Providence Health, 1081 Burrard St, Vancouver, British Columbia V6Z 1Y6 Canada; 4 Department of Medicine, University of British Columbia, Suite 400-13450 102 Ave, Surrey, British Columbia V3T 5X4 Canada

**Keywords:** Oral step down, Antibiotics, *E. coli*, Gram negative bacteremia, Urinary tract infection

## Abstract

**Background:**

Literature is scarce regarding oral step down to beta-lactams in bacteremic urinary tract infections. Oral fluoroquinolones are an accepted and common step down for bacteremic urinary tract infections; however, their use is associated with mounting safety concerns. We compared clinical cure in patients with *E. coli* bacteremic urinary tract infections who were stepped down to oral beta-lactams compared to oral fluoroquinolones.

**Methods:**

This multicentre retrospective cohort study included patients with first positive concurrent urine and blood cultures from January 2016 to December 2016. Patients were included if they received empiric intravenous beta-lactam therapy with step down to either oral beta-lactam or fluoroquinolone for treatment completion. The primary outcome was clinical cure. Secondary outcomes were length of hospitalization, all-cause mortality and *C. difficile* infection. Multivariate analysis and propensity score were used to control for confounding.

**Results:**

A total of 207 patients were identified with bacteremic *E.coli* urinary tract infections. Clinical cure was achieved in 72/77 (94%) in the oral beta-lactam group versus 127/130 (98%) in the oral fluoroquinolone group (absolute difference − 4.2, 95% confidence interval [CI] -10.3 to 1.9%, *p* = 0.13). The adjusted odds ratio (OR) for clinical cure with oral beta-lactams was 0.31 (95% CI 0.05–1.90, *p* = 0.21); propensity score adjusted analysis showed a similar result. There was no statistically significant difference in secondary outcomes.

**Conclusions:**

Oral beta-lactams appear to be a safe and effective step down option in bacteremic *E. coli* urinary tract infections compared to oral fluoroquinolones.

## Summary

Identifying effective alternatives to fluoroquinolones is important given mounting recent safety concerns. This retrospective study finds no difference in clinical outcomes for oral beta-lactam step down in bacteremic *E. coli* urinary tract infections compared to oral fluoroquinolones.

## Background

Pyelonephritis is a common indication for antibiotic use with an incidence of 5–15 cases per 10,000 population [1]. *E. coli* is the most common causative organism, responsible for up to 80% of cases [1, 2]. Treatment guidelines for pyelonephritis have historically favoured use of fluoroquinolones for oral therapy [3]. Oral beta-lactams are thought to be less effective. However, increasing resistance among uropathogens like *E. coli* to fluoroquinolones necessitate a reassessment of the role of oral beta-lactams in management of pyelonephritis. Moreover, regulatory agencies have released multiple recent warnings about the underappreciated harms of fluoroquinolones [4–10].

In pediatric patients with pyelonephritis, oral beta-lactams have already been established as a safe and effective treatment option. Canadian guidelines recommend that oral antibiotics be used as initial management of pyelonephritis, unless the child is toxic or has known structural urologic abnormalities [11].

Oral beta-lactam step down in pyelonephritis was reported routinely during randomized trials in the 1990’s evaluating pyelonephritis management in pregnancy [12–15]. Only one of these trials directly compared intravenous to oral beta-lactam therapy, finding no difference in clinical success; however, bacteremia was excluded [12]. Several further trials in non-pregnant women with pyelonephritis have shown equivalent efficacy of oral beta-lactams in step down compared with ongoing intravenous therapy [16, 17]. Bacteremia was uncommon in these trials (20–25%), raising doubts about whether results could be extrapolated to patients with bacteremic urinary tract infections. Moreover, very little has been published in treatment of adult males with bacteremic pyelonephritis.

We sought to compare the clinical outcomes of adult patients with urinary tract infection and *E. coli* bacteremia who received oral beta-lactam step down compared to oral fluoroquinolone following initial empiric intravenous beta-lactam therapy.

## Methods

### Study design

We conducted a multicentre retrospective cohort study including patients with first positive concurrent blood and urine cultures for *E. coli* from January 1, 2016 to December 31, 2016 within Fraser Health, which serves 1.8 million people in British Columbia, Canada. The study was approved by Fraser Health Research Ethics Board (FHREB File #: 2017–015).

Patients with *E. coli* positive blood and urine cultures were identified from an electronic microbiology database. Patients were included in this study if they were 18 years or older and had both blood and urine cultures positive for *E. coli* within 72 h of each other. Patients were only included if they received empiric intravenous beta-lactam therapy and followed by step down to oral beta-lactam or oral fluoroquinolone for treatment completion. Timing and agent of step down therapy was at the discretion of the treating physician. Patients who did not survive for at least 72 h after first positive blood culture were excluded.

Patients were also excluded for the following reasons: non-urinary source of *E. coli* bacteremia as documented by the treating physician; treatment for a concomitant infection; presence of complicated pyelonephritis identified within first 7 days of therapy (emphysematous pyelonephritis, renal parenchymal abscess, perinephric abscess, renal papillary necrosis); prostatitis; extended spectrum beta-lactamase producing, AmpC, or carbapenem-resistant *E. coli*; HIV with CD4 < 200; neutropenia with ANC < 0.5; or renal transplant.

Demographic, clinical and outcome data were acquired from electronic medical records. Charlson co-morbidity index and Pitt bacteremia score data were collected at baseline. Sepsis and septic shock was defined using the Sepsis-3 international consensus definitions [18]. Infections were classified as community-acquired healthcare-associated infections if any of the following criteria were met: Living in a long-term care facility or nursing home, receiving outpatient intravenous therapy within the past 30 days, attending a hospital or hemodialysis clinic within the past 30 days or being admitted for more than 2 days within the past 90 days. A full list of definitions can be found in Additional file 1.

### Outcome measures

The primary outcome was clinical cure defined by meeting all of the following criteria: resolution or improvement of symptoms during treatment, no recurrence of symptoms or signs of urinary tract infection within 30 days, and no discontinuation or change of treatment because of worsened or persistent symptoms or occurrence of adverse events.

Secondary outcomes included length of hospitalization following first positive blood culture, all-cause mortality within 30 days of first positive blood culture, and *C. difficile* positive stool sample within 30 days of first positive blood culture.

### Microbiology methods

Blood and urine cultures were processed by a centralized clinical microbiology laboratory located within Fraser Health. Antibiotic susceptibility was determined by the Vitek 2 System (bioMérieux, Laval, Quebec). Clinical and Laboratory Standards Institute breakpoints were used to determine antimicrobial susceptibility breakpoints (2016 M100 26th edition).

### Statistical analysis

Categorical variables were analyzed using Pearson chi-squared or Fisher’s exact test. Continuous variables were analyzed using Mann Whitney U test. Statistical significance for all tests was set at a *p*-value of less than 0.05.

Association between antibiotic therapy (oral beta-lactam or oral fluoroquinolone) and clinical cure was modelled using logistic regression and expressed as odds ratios (ORs) and 95% confidence intervals (CI). Additional adjustment for covariates with a *P* value of < 0.20 on univariate analysis were included in the adjusted logistic regression model.

Further analysis using propensity score was performed to account for potential residual confounding. We constructed a propensity score for receiving antibiotic therapy (oral beta-lactam or oral fluoroquinolone) as the dependent variable using logistic regression modelling. Covariates included in generating the propensity score included age, sex, diabetes mellitus, chronic kidney disease, heart conditions, immune compromise, recurrent UTI risk factors (history of recurrent UTI, anatomical or functional urinary tract abnormalities, or nephrolithiasis), Pitt bacteremia score, sepsis, beta-lactam allergy, and duration of intravenous therapy. A final analysis using logistic regression to assess for association between antibiotic therapy and clinical cure was performed using propensity score as a single covariate. Statistical analyses were performed using STATA IC version 13.1 and RStudio version 1.2.503.

## Results

There were 207 patients who met eligibility criteria during the study period. One hundred and thirty patients (62.8%) were stepped down to an oral fluoroquinolone, while 77 (37.2%) were stepped down to an oral beta-lactam. Baseline clinical and demographic data are presented in Table 1. Patients stepped down to an oral beta-lactam were more likely to have acquired the UTI in a community acquired health-care associated setting (26% vs 11.5%) and less likely in a community acquired setting (68.8% vs 84.6%). All other baseline factors were similar between groups, including indicators for severity of infection such as sepsis or septic shock, ICU admission, and Pitt bacteremia score. The median number of days of intravenous treatment was similar between patients stepped down to oral beta-lactam compared to oral fluoroquinolone (5 vs 5 days, *p* = 0.20), as was oral treatment duration (7 vs 7 days, *p* = 0.38), and total treatment duration (14 vs 14 days, *p* = 0.91).

**Table 1:** Baseline characteristics of patients

	Oral fluoroquinolone step down (***n*** = 130)	Oral beta-lactam step down (***n*** = 77)	***p***-value
Age, median, years (IQR)	70.5 (56.25–80.75)	71 (54–80)	0.72
Sex			0.92
Female	92 (71)	54 (70)	
Male	38 (29)	23 (30)	
Comorbidities			
Diabetes Mellitus	29 (22)	20 (26)	0.55
Heart Conditions	75 (58)	43 (56)	0.8
Chronic Kidney Disease	10 (8)	3 (4)	0.28
Peripheral Vascular Disease	6 (5)	4 (5)	0.85
Asthma or COPD	14 (11)	7 (9)	0.7
Liver Cirrhosis	1 (1)	2 (3)	0.56
Recurrent UTI Risk factors	38 (29)	22 (29)	0.92
IVDU	5 (4)	0 (0)	0.16
Hepatitis C	1 (1)	1 (1)	1.0
HIV	0 (0)	0 (0)	1.0
Immunocompromised	5 (4)	4 (5)	0.65
Charlson comorbidity index, median (IQR)	1 (0–2)	1 (0–3)	0.96
Catheter associated UTI	6 (5)	4 (5)	0.85
Hospital admission	101 (78)	62 (81)	0.63
ICU admission	9 (7)	5 (6)	0.91
Sepsis	31 (25)	25 (33)	0.2
Septic shock	8 (6)	5 (6)	0.92
Pitt bacteremia score, median (IQR)	0.5 (0–1)	1 (0–1)	0.53
Site of acquisition			0.02
Community-acquired	110 (85)	53 (69)	
Community-acquired health-care associated	15 (12)	20 (26)	
Hospital-acquired	5 (4)	4 (5)	
Beta-lactam allergy	15 (12)	7 (9)	0.58
Inadequate empiric treatment	1 (1)	1 (1)	1.0
Stable at oral switch	125 (96)	74 (96)	0.99
Duration of intravenous treatment, median, days (IQR)	5 (3–7)	5 (3–7)	0.20
Duration of oral treatment, median, days (IQR)	7 (7–10)	7 (5.75–10)	0.38
Total treatment duration, median, days (IQR)	14 (12–14)	14 (11–14)	0.91

Data are number (percentage) of patients unless otherwise indicated

*Abbreviations*: *COPD* chronic obstructive pulmonary disease, *ICU* intensive care unit, *IVDU* intravenous drug use, *UTI* urinary tract infection;

The primary outcome of clinical cure was achieved in 127 patients (98%) in the oral fluoroquinolone group and in 72 patients (94%) in the oral beta-lactam group (absolute difference − 4.2, 95% CI, − 10.3 to 1.9%, *p* = 0.13) (Table 2).

**Table 2:** Thirty-day outcomes for patients

	Oral fluoroquinolone step down (***n*** = 130)	Oral beta-lactam step down (***n*** = 77)	***p***-value
Primary Outcome			
Clinical cure	127 (98)	72 (94)	0.13
Secondary Outcomes			
30-day mortality	1 (1)	0 (0)	0.43
CDI	1 (1)	1 (1)	1
Length of hospitalization, median, days (IQR)	6 (3.25–9)	6 (4–10)	0.43

Data are number (percentage) of patients unless otherwise indicated

*Abbreviation*: *CDI Clostridioides difficile* infection

Unadjusted analysis demonstrated no significant difference between oral beta-lactam and oral fluoroquinolone groups for clinical cure (OR 0.34; 95% CI, 0.08–1.47, *p* = 0.15) (Table 3). Results were similar using multivariate adjustment (adjusted OR, 0.31; 95% CI, 0.05–1.90, *p* = 0.2) and propensity scoring adjustment (OR, 0.31; 95% CI, 0.07–1.38, *p* = 0.12). Female sex was not associated with higher clinical cure (OR 2.49; 95% CI, 0.60–10.30, *p* = 0.21).

**Table 3:** Univariate and multivariate analysis for thirty-day clinical cure in *E. coli* bacteremic urinary tract infections

Variable	Unadjusted Analysis		Multivariate Analysis	
	OR (95% CI)	***p***-value	OR (95% CI)	***p***-value
Age (per year)	0.98 (0.93–1.02)	0.30	…	…
Sex (female)	2.49 (0.60–10.30)	0.21	…	…
DM	0.29 (0.07–1.22)	0.09	0.50 (0.08–3.04)	0.45
CKD	0.18 (0.03–0.97)	0.046	0.28 (0.03–3.06)	0.30
Heart Conditions	0.18 (0.02–1.49)	0.11	0.38 (0.03–4.43)	0.44
Asthma/COPD	0.78 (0.09–6.69)	0.82	…	…
Immunocompromised	0.29 (0.03–2.68)	0.28	…	…
Recurrent UTI risk factors	0.12 (0.02–0.63)	0.01	0.15 (0.02–0.96)	0.045
CA-UTI	0.33 (0.04–2.99)	0.33	…	…
Charlson (per unit)	0.69 (0.52–0.92)	0.01	0.71 (0.41–1.21)	0.20
Pitt (per unit)	0.72 (0.49–1.04)	0.08	0.66 (0.39–1.10)	0.11
Sepsis	0.61 (0.14–2.62)	0.50	…	…
Septic Shock	0.45 (0.05–3.95)	0.47	…	…
ICU admission	0.49 (0.05–4.28)	0.52	…	…
Beta-lactam allergy	0.34 (0.06–1.77)	0.20	0.14 (0.02–1.14)	0.07
IV therapy duration	0.83 (0.67–1.03)	0.09	0.88 (0.65–1.18)	0.40
Total duration	1.01 (0.80–1.27)	0.96	…	…
Oral beta-lactam step down	0.34 (0.08–1.47)	0.15	0.31 (0.05–1.90)	0.21

*Abbreviations*: *CA-UTI* catheter-associated urinary tract infection, *CKD* chronic kidney disease, *CI* confidence interval, *COPD* chronic obstructive pulmonary disease, *DM* diabetes mellitus, *ICU* intensive care unit, *IV* intravenous, *OR* odds ratio, *UTI* urinary tract infection

The median length of hospitalization was 6 days in both groups (*p* = 0.43). There was one mortality in the oral fluoroquinolone group, and one case of *C. difficile* in each group; neither of these secondary outcomes were statistically significant.

Patients stepped down to an oral fluoroquinolone were all prescribed ciprofloxacin (Table 4). Conversely, a variety of antibiotics were used as step down for those given an oral beta-lactam, including amoxicillin, amoxicillin-clavulanate, cephalexin, cefuroxime, and cefixime.

**Table 4:** Oral antibiotic step down regimens

	Antibiotic	Regimen	Frequency (%)
Oral fluoroquinolone step down (*n* = 130)	Ciprofloxacin	500 mg po BID	114 (87.7)
		500 mg po daily	5 (3.9)
		250 mg po BID	5 (3.9)
		750 mg po BID	2 (1.5)
	Ciprofloxacin ER	1000 mg po daily	3 (2.3)
		500 mg po daily	1 (0.8)
Oral beta-lactam step down (*n* = 77)	Cefixime	400 mg po daily	24 (31.2)
	Cephalexin	500 mg po QID	19 (24.7)
		1000 mg po QID	1 (1.3)
	Amoxicillin-clavulanate	875 mg po BID	10 (13.0)
		500 mg po TID	7 (9.1)
		500 mg po BID	2 (2.6)
	Amoxicillin	500 mg po TID	5 (6.5)
		500 mg po BID	2 (2.6)
	Cefuroxime	500 mg po BID	7 (9.1)

Data are number (percentage) of patients unless otherwise indicated

*Abbreviation*: *ER* extended release, *BID* two times a day, *TID* three times a day, *QID* four times a day

## Discussion

Our results suggest that oral beta-lactams are a safe and effective step down for patients with *E. coli* bacteremic urinary tract infections. At baseline, the two treatment groups had similar co-morbidities, severity of infection and duration of intravenous treatment. In this population of bacteremic patients, there was no difference in rates of clinical cure or length of hospitalization. Approximately 30% of patients in our study were male with no significant difference in clinical cure rate compared to females. Mortality and *C. difficile* infection were infrequent occurrences, with no difference between the groups.

This study isolated a group of bacteremic patients with pyelonephritis to demonstrate the suitability of oral beta-lactam step down and adds to a growing body of literature with similar conclusions. A randomized controlled trial by Sanchez et al. compared daily intravenous ceftriaxone to single dose intravenous ceftriaxone followed by oral cefixime, both to total 10 days of treatment [16]. Their study included 144 women with uncomplicated pyelonephritis*. E. coli* was the responsible organism in nearly all cases and 25% of patients had bacteremia. There was no difference in clinical cure between groups (91% vs 92% respectively). Another randomized controlled trial by Monmaturapoj et al. evaluated daily intravenous ceftriaxone to 3 days of intravenous ceftriaxone followed by oral cefditoren, both to total 10 days of treatment [17]. Their study included 82 patients with pyelonephritis, only three of whom were male. *E. coli* was identified in 84% of cases and 21% of patients had bacteremia. Again, no difference in clinical cure rate was found (95% vs 100% respectively). Both of these randomized controlled trials were limited by the small number of male patients and minority of cases with bacteremia.

A recent observational study by Tamma et al. showed similar 30-day mortality in a cohort of hospitalized patients with *Enterobacteriaceae* bacteremia who received oral step down therapy compared to ongoing intravenous therapy [19]. Nearly half the patients in their study were male. A wide range of infections were included, with urinary tract infection comprising 36.5%. However, fluoroquinolones were the most commonly used oral step down therapy (70%), while oral beta-lactams were only used in a minority (16.5%). This reflects the widespread practice of avoiding oral beta-lactams in step down therapy for gram negative bacteremia.

Mercuro et al. compared fluoroquinolones to beta-lactams in oral step down for *Enterobacteriaceae* bacteremia [20]. Over 50% of patients were male. *E. coli* was the dominant organism (71%), with approximately 70% having a urinary tract infection source. No difference in clinical success was noted between the two groups. However, their study included only admitted patients. Over 20% of patients in our study were managed as outpatients, without hospital admission. Furthermore, including a broad range of infections may mask differences between individual subgroups (e.g., urinary tract infections). As a homogenous patient population of bacteremic urinary tract infections our study is not affected by this limitation.

A recent meta-analysis by Punjabi et al. found no difference in all-cause mortality between oral beta-lactam and oral fluoroquinolones in step down of *Enterobacteriaceae* bacteremia [21]. Oral beta-lactams appeared to have higher rates of infection recurrence compared to fluoroquinolones. The authors hypothesize that under-dosing of beta-lactams may be contributing to this finding. Including a wide range of source infections confounds this interpretation.

The impetus to limit fluoroquinolone prescribing is growing. Fluoroquinolone prescribing is an established risk for *C. difficile* infection, both at the individual and community level [22, 23]. Multiple statements have been issued by Health Canada, the United States Food and Drug Administration, and the European Medicines Association warning about serious and underappreciated side effects [4–10]. For fluoroquinolone prescribing to be curbed, other therapeutic options with similar efficacy and improved safety profile need to be identified. Furthermore, despite the side effects of fluoroquinolones, they fulfil a critical role as oral therapy for difficult to treat gram-negative infections due to *Pseudomonas* or *Acinetobacter*. Limiting fluoroquinolone use to these infections where other suitable oral alternatives do not exist is prudent antimicrobial stewardship.

Fluoroquinolones have long been regarded as an effective agent for bacteremic infections. Pharmacologic factors including high oral bioavailability, convenient dosing, and ability to attain dosing targets have supported this practice. This is supported by a large body of controlled literature and extensive real life clinical experience. Oral beta-lactams do not share the same historical confidence in serious invasive infections including bacteremia. Pharmacokinetic concerns including lower oral bioavailability and more frequent dosing are reason for pause. However, recent randomized controlled trials using oral beta-lactams in treatment of infective endocarditis and complex orthopedic infections challenge these historical dogmas [24, 25].

Our study has several limitations. First, its retrospective design limits our ability to control for confounders to those that were collected. Second, selection bias could have been an issue, as unaccounted patient factors may have led a physician to choose a certain oral step down option, or not to step down to oral therapy at all. The use of propensity score analysis can partly account for this confounding. Third, some relevant outcomes may have been missed. Data were abstracted from the Health Authority electronic medical record, thus potentially relevant outcomes only captured in outpatient records were not accessible. Fourth, the convenience sample of 1 year resulted in a limited sample size. This study may be underpowered to identify a difference between treatment arms where one truly does exist. Fifth, our study excluded complicated cases (e.g., renal abscess) and prostatitis. Our results should not be extrapolated to the whole population of bacteremic urinary tract infections. Lastly, both groups received a median of 5 days of intravenous therapy and 14 days of total treatment. This longer treatment duration may have reduced the chance to find a difference between the groups.

## Conclusion

Future research is needed to further establish the safety, efficacy, and optimal duration of oral step down therapy for bacteremic urinary tract infections, in particular with oral beta-lactams. While observational studies can corroborate our findings, randomized controlled trials would provide clinicians the greatest confidence in using oral beta-lactam therapy as step down for bacteremic urinary tract infections.

In conclusion, oral beta-lactam step down in the setting of urinary tract infections with *E. coli* bacteremia had a similar clinical cure rate to that of oral fluoroquinolone step down. Given the potential adverse effects associated with fluoroquinolones, beta-lactams present a safe and efficacious alternative oral step-down agent.

## Supplementary information

**Additional file 1.** Definitions of terms used to collect data

## Data Availability

The datasets used during the current study are available from the corresponding author on reasonable request.

## Abbreviations

ORs: Odds ratios
CI: Confidence intervals
UTI: Urinary tract infection
ICU: Intensive care unit
COPD: Chronic obstructive pulmonary disease
IVDU: Intravenous drug use
CDI: *Clostridioides difficile* infection
CA-UTI: Catheter-associated urinary tract infection
CKD: Chronic kidney disease
DM: Diabetes mellitus
IV: Intravenous
ER: Extended release
BID: Two times a day
TID: Three times a day
QID: Four times a day
