# Supplementary material for: Oral beta-lactam step down in bacteremic E. coli urinary tract infections
Source: BMC Infect Dis. 2020 Oct 21;20:785. doi: 10.1186/s12879-020-05498-2 (PMC7576740; doi:10.1186/s12879-020-05498-2)
Supplement: Supplementary file 1 — Additional file 1. Definitions of terms used to collect data [file 12879_2020_5498_MOESM1_ESM.docx]

Additional file 1 - Definitions

**Definition of terms used to collect data**

| **Term** | **Definition** |
| --- | --- |
| Bacteremia | Bacteria isolated from blood cultures |
| Pyelonephritis | Infection of the upper urinary tract |
| Bacteremic pyelonephritis | Bacteria isolated from blood cultures in the context of pyelonephritis |
| Urinary tract infection | Infection of any part of the urinary tract |
| Catheter associated urinary tract infection | Urinary tract infection occurring in a person whose urinary tract is currently catheterized or has been catheterized within the previous 48 hours |
| Sepsis | Meets 2 out of 3 criteria: Respiratory rate ≥22/min, altered mentation and systolic blood pressure ≤100 mm Hg |
| Septic shock | Sepsis with persistent hypotension requiring vasopressors to maintain MAP > 65 mmHg |
| Community-acquired | Blood culture obtained in outpatients or inpatients admitted <48 hours who do not meet criteria for healthcare-associated |
| Community-acquired healthcare-associated | Blood culture obtained in outpatients or inpatients admitted <48 hours who meet one of the following criteria: Long-term care facility or nursing-home residents, received outpatient IV therapy within the past 30 days, attended a hospital or hemodialysis clinic within the past 30 days, admitted for >2 days in the preceding 90 days |
| Hospital-acquired | Blood culture obtained ≥48 hours after hospital admission. |
| Clinical cure | Meets all of the following criteria: Resolution or improvement of symptoms during treatment, no recurrence of symptoms or signs of urinary tract infection within 30 days, and no discontinuation or change of treatment because of worsened or persistent symptoms or occurrence of adverse events |

|  |
| --- |
